# Systematic Review of Robotic‐Assisted Peripheral and Central Lymphatic Surgery

**DOI:** 10.1002/jso.27866

**Published:** 2024-09-10

**Authors:** Imholz Carlotta, Grünherz Lisanne, Lindenblatt Nicole

**Affiliations:** ^1^ Department of Plastic and Hand Surgery University Hospital Zurich Zurich Switzerland; ^2^ Faculty of Medicine University of Zurich Zurich Switzerland

**Keywords:** central lymphatic surgery, lymphatic surgery, robotic‐assisted central lymphatic surgery, robotic‐assisted lymphatic surgery, robotic‐assisted microsurgery, thoracic duct–vein anastomosis

## Abstract

**Background:**

Robotic‐assisted lymphatic reconstruction has gained increasing interest over the past few years.

**Objectives:**

The aim of this study was to systematically investigate the benefits of robotic‐assisted lymphatic surgery based on currently published literature.

**Methods:**

A systematic review evaluating the feasibility, surgical aspects, and both objective and subjective improvements in patients with impairment of the peripheral or central lymphatic system was performed according to the PRISMA guidelines. The review was registered on PROSPERO.

**Results:**

The literature search yielded 328 articles after the removal of duplicates, followed by a full‐text review of the 29 articles, out of which a total of 11 relevant articles were deemed eligible. Among these, seven used a retrospective design and four a prospective design. All studies included confirmed the feasibility of robotic‐assisted lymphatic surgery and reported promising results concerning both technical aspects and patient‐related outcomes. However, currently, only a limited number of studies directly compare the robotic‐assisted approach to the manual approach, and these studies have limited statistical analyses.

**Conclusion:**

Despite the heterogeneous measurands, all studies showed the feasibility of robotic‐assisted lymphatic surgery, and seven provided promising data on patient‐related outcomes. Additional studies are needed to further identify future directions in robotic‐assisted lymphatic surgery.

## 1 Introduction

Robotic‐assisted lymphatic reconstruction has gained increasing interest with a growing body of literature over the past few years. The introduction of two dedicated microsurgical robotic platforms in 2017 and 2021 allowed the performance of a variety of robotic‐assisted procedures on the peripheral and central lymphatic system, showing promising results regarding feasibility, dexterity, and access to different anatomical levels [1, 2, 3].

Before this, the first‐in‐human experience with robotic‐assisted microsurgery was made in 2006, when the arterial anastomosis of a free transverse rectus abdominis myocutaneous flap was performed with the Da Vinci robot (Intuitive Surgical Inc., Sunnyvale, USA) [4]. This was followed by a few other microsurgical cases such as robotic‐assisted brachial plexus surgery and vascular anastomosis in head and neck reconstruction [5, 6, 7]. Although these cases showed the feasibility of using the Da Vinci robot in microsurgery, its drawbacks such as the absence of sophisticated microsurgical instrumentation and reduced image magnification in comparison to a surgical microscope outweighed its benefits [6]. This indicated the necessity to develop dedicated microsurgical robotic platforms to overcome the limitations of the Da Vinci system.

Two microsurgical robots have been developed and are in clinical use to date: the MUSA (MicroSure, Eindhoven, the Netherlands) and the Symani Surgical System (Medical Microinstruments [MMI], Jacksonville, FL, USA). In 2017, the MUSA robot was used for the first‐in‐human robotic‐assisted lymphovenous anastomosis (LVA) in patients with breast cancer–related lymphedema, marking its introduction into clinical use [1]. The initial application of the Symani Surgical System in lymphatic reconstruction was reported in 2021, where robotic‐assisted LVAs, vascularized lymph node transfers (VLNTs), and lympholymphatic anastomoses (LLAs) were performed in several patients for the treatment of lymphedema or as a preventive measure following tumor resection in the groin [2]. Recently, the usage of the Symani Surgical System in lymphatic surgery has extended from interventions on the peripheral lymphatic system to those on the central lymphatic system. In 2023, the Symani Surgical System was used for central lymphatic reconstruction for the first time by performing an anastomosis of the ovarian vein to an abdominal cyst of the central lymphatic system [3]. This initial success in robotic‐assisted central lymphatic reconstruction was followed by a case series of several robotic‐assisted thoracic duct–vein anastomoses (TDVAs) in 2024 [8, 9].

So far, different studies reported promising results regarding improved precision through motion scaling and the elimination of physiologic tremor as well as facilitated access to deep‐lying structures [10]. Yet, the implementation of robotic assistance in lymphatic surgery is still in its early stage, which is partly due to a lack of literature comparing robotic‐assisted and manual techniques and to disadvantages such as longer operating times and higher costs that need to be further evaluated [11].

This study aims to investigate the benefits of robotic‐assisted lymphatic surgery based on various outcomes and to conclude what should be considered in the future to overcome current limitations. Based on this, we performed a systematic review of the existing literature on robotic‐assisted lymphatic surgery, evaluating the feasibility, surgical aspects, and both objective and subjective improvements in patients with impairment of the peripheral or central lymphatic system.

## 2 Methods

A systematic review was conducted following the Preferred Reporting Items for Systematic Reviews and Meta‐Analysis (PRISMA) guidelines, with an initial review protocol established and registered on PROSPERO, the international prospective register of systematic reviews (CRD42024555916). The literature search was conducted on June 4, 2024 in the following databases: MEDLINE, EMBASE, Cochrane, Web of Science Core Collection, Web of Science Preprint Citation Index, ClinicalTrials.gov, WHO International Clinical Trials Registry Platform, and LILACS. The language was restricted to English and German. Further, there was a restriction to in‐human studies, but there was no time restriction. The following search terms including related terms were used: lymphedema, lymphovenous anastomosis, lymph node transplantation, thoracic duct–vein anastomosis, robot‐assisted microsurgery, symani, and musa. A detailed description of the search strategy can be found in File S1.

Two reviewers independently screened all references identified in the search based on title and abstract. Subsequently, the resulting articles were assessed for eligibility through full‐text reading. Due to the limited number of studies on robotic‐assisted lymphatic reconstruction, inclusion criteria encompassed all clinical studies in which patients underwent a robotic‐assisted surgical intervention on the peripheral or central lymphatic system. Reviews, editorials, cadavers, and animal studies, as well as conference abstracts, were excluded.

For the data extraction of the included studies, the following quantitative and qualitative variables were recorded: year of publication, type of study, robotic system, interventions, total number of patients undergoing lymphatic surgery, number of patients with robotic assistance, number of anastomoses, mean time for anastomosis, mean number of stitches, mean follow‐up time, patency, objective and subjective improvement, and adverse events. The variables were extracted separately for anastomoses performed with robotic assistance and those done with manual technique. In one case, data were requested to identify the exact number of lymphatic and non–LLAs, as the study contained both [12].

A narrative synthesis with descriptive statistics of the results of the included studies was conducted. No inferential statistical analysis was performed as different studies used different metrics to describe the results and both quantitative and qualitative data were included.

## 3 Results

**Figure 1:**
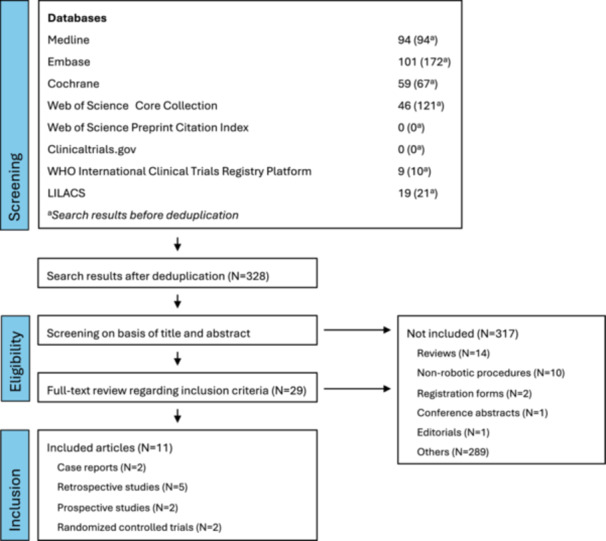
Flowchart of the selection process of articles.

The literature search resulted in the identification of 328 articles after the removal of duplicates. Figure 1 shows the process of article inclusion and exclusion based on title and abstract in the first step, followed by a full‐text review of the remaining 29 articles, out of which a total of 12 relevant articles were deemed eligible. However, two of these studies described the same case [13, 14]; therefore one was excluded, resulting in the inclusion of a total of 11 articles. Among the included studies, seven used a retrospective design and four a prospective design. The latter includes both a randomized controlled pilot trial and a 1‐year follow‐up involving the same patient cohort [1, 15]. Across all studies, we included 144 patients, excluding the patient cohort of the 1‐year follow‐up and patients receiving non‐lymphatic surgery. Of these, 126 patients have received different types of robotic‐assisted lymphatic reconstruction including LVA, LLA, VLNT, TDVA, and anastomosis of a vein to a lymphatic cyst of the central lymphatic system. The majority of the included studies (*n* = 8) used the Symani Surgical System, while the remaining three articles worked with the MUSA robot. Table 1 provides an overview of all articles included in our review.

**Table 1:** Overview of all included studies.

Publication	Study type	Robotic system	Intervention	Localization	Total patients (*n*)	Patients with robotic assistance (*n*)
Lindenblatt et al. [2]	Retrospective	Symani Surgical System	LVA, VLNT, LLA, MLL	Peripheral	5	5
Barbon et al. [12]	Retrospective	Symani Surgical System	LVA, VLNT, LLA	Peripheral	18	18
Weinzierl et al. [10]	Retrospective	Symani Surgical System	LVA, VLNT, liposuction	Peripheral	8	8
Grünherz et al. [3]	Case report	Symani Surgical System	Anastomosis of the ovarian vein to the lymphatic cyst of the central lymphatic system	Central	1	1
Strübing et al. [14]	Prospective	Symani Surgical System	LVA	Peripheral	1	1
von Reibnitz et al. [16]	Retrospective	Symani Surgical System	LVA, VLNT, LLA, liposuction	Central and peripheral	67	67
Lilja, Thomsen, and Sørensen [17]	Case report	Symani Surgical System	LVA, excision of lymphocele	Peripheral	1	1
Weinzierl et al. [8]	Retrospective	Symani Surgical System	LVA, TDVA, vein–lymphatic cyst anastomosis, MLL	Central	11	5
van Mulken et al. [1]	Randomized pilot trial	MUSA robot	LVA	Peripheral	20	8
van Mulken et al. [15]	Randomized pilot trial— 1‐year follow‐up	MUSA robot	LVA	Peripheral	17	6
Reilly et al. [18]	Prospective	MUSA‐2 robot	LVA	Peripheral	12	12

Abbreviations: LLA, lympholymphatic anastomosis; LVA, lymphovenous anastomosis; MLL, microscopic lymphatic ligation; TDVA, thoracic duct–vein anastomosis; VLNT, vascularized lymph node transfer.

### 3.1 Surgical Details

**Figure 2:**
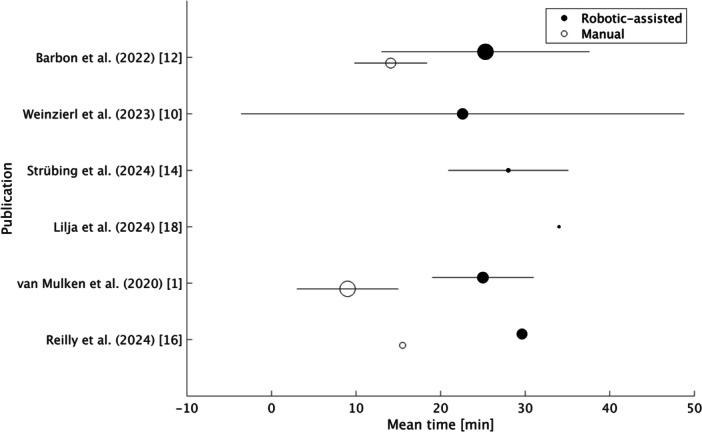
Scatter plot indicating the mean time for robotic‐assisted and manual anastomoses for each study reporting either of these values. Filled circles represent robotic‐assisted anastomoses, whereas empty circles represent manual anastomoses. The circle size indicates the number of anastomoses. The horizontal lines show the standard deviations stated in the publications.

A total of ≥ 186 robotic‐assisted and ≥ 58 manual anastomoses (LVAs, LLAs, VLNTs, TDVAs, and vein–cyst anastomosis) have been reported across the included studies (excluding non–LLAs). In five studies, all patients received solely robotic‐assisted lymphatic reconstruction, whereas in the six remaining studies, patients received either robotic‐assisted or manual anastomosis or a combination of both. The mean time to perform a robotic‐assisted anastomosis was stated in seven studies, with a weighted average of 25.68 min (range 22.6–29.625 min) based on the number of anastomoses. In comparison, the weighted average of the mean time to perform a manual anastomosis, as reported in four studies, amounts to 11 min (range 9–15.5 min). Figure 2 shows the mean time for both robotic‐assisted and manual anastomoses for each study reporting either of these values.

Barbon et al. and van Mulken et al. both contrasted the mean time for robotic‐assisted and manual anastomoses, showing a significantly longer anastomotic time for robotic‐assisted anastomoses, with a difference in mean time of 11.2 min (*p* < 0.01) with the Symani Surgical System and 16 min with the MUSA robot (*p* < 0.001) [1, 12]. However, apart from 18 patients with robotic‐assisted lymphatic surgery, the study of Barbon et al. additionally included four patients who underwent non‐lymphatic robotic‐assisted procedures such as free flaps and nerve coaptation, which are also represented in the data on mean anastomotic time and mean number of stitches stated in Table 2. Furthermore, following a robotic‐assisted LVA, Reilly et al. performed a second LVA in eight patients using either the MUSA robot (*n* = 4) or manual technique (*n* = 4) [18]. The mean anastomotic time amounted to 22.5 min for the robotic‐assisted and 15.5 min for the manual second LVA.

**Table 2:** Surgical details.

Publication	Robotic anastomoses (*n*)	Manual anastomoses (*n*)	Mean time for robotic anastomoses (min)	Mean time for manual anastomoses (min)	Mean number of stitches (robotic)	Mean number of stitches (manual)
Lindenblatt et al. [2]	10 (LVA/LLA 8, artery 2)	8	NA	NA	NA	NA
Barbon et al. [12]	28 (LVA 20, LLA 1, artery 7)	11 (LVA 8, artery 3)	25.3 ± 12.3, range 10–59; LVA 1st group: 23.9 ± 6.8, 2nd group: 16.3 ± 6.1 (*p* < 0.05)	14.1 ± 4.3 (*p* < 0.01), range 8–35	6.8 ± 1.8	6.1 ± 1.5 (*p* = n.sig.)
Weinzierl et al. [10]	13 (VLNT 9, LVA 4)	NA	22.6 ± 26.2, range 11–59	NA	7.9 ± 1.4	NA
Grünherz et al. [3]	1	NA	NA	NA	NA	NA
Strübing et al. [14]	2	NA	28.0 ± 7.1	NA	5 ± 0	NA
von Reibnitz et al. [16]	100 (LVA 53, VLNT 44, LLA 3)	NA	NA	NA	NA	NA
Lilja, Thomsen, and Sørensen [17]	1	NA	34	NA	NA	NA
Weinzierl et al. [8]	≥ 5 (LVA ≥ 1, TDVA ≥ 2, vein‐cyst‐anastomosis 1)	≥ 9 (LVA ≥ 5, TDVA ≥ 4)	NA	NA	NA	NA
van Mulken et al. [1]	14	26	25 ± 6, range 16–33	9 ± 6 (*p* < 0.001), range 4–36	NA	NA
van Mulken et al. [15]	14	26	25 ± 6, range 16–33	9 ± 6 (*p* < 0.001), range 4–36	NA	NA
Reilly et al. [18]	> 12	> 4	29.625; 1st anastomosis: 32, 2nd anastomosis: 22.5 (*n* = 4)	2nd anastomosis: 15.5 (*n* = 4)	4.5	NA

Abbreviations: LLA, lympholymphatic anastomosis; LVA, lymphovenous anastomosis; NA, not available; n. sig., not significant; TDVA, thoracic duct–vein anastomosis; VLNT, vascularized lymph node transfer.

One study differentiated between the mean times for the first and second anastomosis in patients receiving multiple anastomoses [18]. The study reported a shorter duration for the performance of the second robotic‐assisted anastomosis, with a difference in time of 9.5 min between the first and second anastomosis. Barbon et al. compared the mean time to perform robotic‐assisted LVA in two consecutive groups, thus investigating the learning curve using a microsurgical robot [12]. A significant improvement (*p* < 0.05) was observed, with a mean time of 23.9 ± 6.8 min in the first patient group and a mean time of 16.3 ± 6.1 min in the second patient group.

Four studies reported the mean number of stitches for robotic‐assisted anastomosis, with a weighted average of 6.49 stitches (range 4.5–7.9 stitches). Only one study compared the mean number of stitches between robotic‐assisted and manual anastomoses [12]. However, there was no significant difference found between the mean number of stitches for robotic‐assisted (6.8 ± 1.8) and manual (6.1 ± 1.5) anastomoses.

The surgical details discussed in this section are listed in Table 2.

### 3.2 Central Lymphatic Surgery

Two studies included reported the successful use of robotic assistance in central lymphatic reconstruction [3, 8]. Weinzierl et al. analyzed the outcome in 11 patients receiving either robotic‐assisted or manual TDVA or vein‐cyst anastomosis [8]. The study stated a regression of symptoms and an improvement of clinical manifestations such as anasarca, chylothorax, and lymphedema in 80% of the patients (*n* = 4) in the robotic‐assisted surgery group and 83.33% (*n* = 5) in the manual surgery group. Grünherz et al. described a case, in which a robotic‐assisted anastomosis between the left ovarian vein and an aneurysmal malformation of the central lymphatic system was performed [3]. This resulted in a complete regression of abdominal pain and circulatory reactions, as well as an increased physical strength at 3 months postoperatively.

### 3.3 Surgical Outcome

The outcome of robotic‐assisted lymphatic reconstruction was described using a variety of measured quantities across the different studies. Table 3 provides an overview of different measurands regarding the surgical outcome and the respective mean follow‐up time.

**Table 3:** Surgical outcome.

Publication	Patency (robotic)	Patency (manual)	Mean follow‐up time (months)	Outcome	Complications
Lindenblatt et al. [2]	100%	100%	NA	NA	NA
Barbon et al. [12]	97.5%	97.5%	NA	NA	NA
Weinzierl et al. [10]	100%	NA	0.5 (*n* = 1); 3 (*n* = 1)	ΔVol UE −25.2%, Δ of excess volume in comparison to unaffected arm −100% (*n* = 1); lymphedema reduction in forearm and hand (*n* = 1)	NA
Grünherz et al. [3]	100%	NA	3	Complete regression of pain, increased physical strength	NA
Strübing et al. [14]	NA	NA	2.17 (66 d ± 27 d)	NA	NA
von Reibnitz et al. [16]	NA	NA	10.1, range 0–26	ΔVol UE (*n* = 6, 86%), mean ΔVol per arm −281 mL (−7.6%) at 3 months; ΔVol LE (*n* = 23, 72%), mean ΔVol per leg −288 mL (−1.4%); complete symptom regression (*n* = 1)	Wound infection of surgical site (*n* = 6, 6.4%; additional wound dehiscence (*n* = 1) and development of lymphocele (*n* = 1)); wound dehiscence (*n* = 1); hematoma (*n* = 1)
Lilja, Thomsen, and Sørensen [17]	100%	NA	1	Complete remission of lymphocele, improved control and functionality of leg	No postoperative complications
Weinzierl et al. [8]	NA	NA	Rob.: 3.6, range 1–7; Man.: 23.1, range 1.5–60	Rob.: regression of clinical manifestations (*n* = 4, 80%); Man.: regression of clinical manifestations (*n* = 5, 83.33%)	NA
van Mulken et al. [1]	100%	100%	3	Rob. (baseline *n* = 8, 3 months *n* = 6): mean UEL index (116 ± 24, 113.01 ± 21), mean lymph‐ICF score: (38 ± 16, 22 ± 16); Man. (baseline *n* = 12, 3 months *n* = 11): mean UEL index (122 ± 20, 125 ± 19), mean lymph‐ICF score (49 ± 16, 29 ± 19)	Suspected erysipelas infection (*n* = 2)
van Mulken et al. [15]	66.6% (*n* = 4, % of patients with ≥ 1 patent anastomosis)	81.8% (*n* = 9)	12 (rob.: 378 d, man.: 376 d)	Rob.: mean UEL index (123.44, *p* = 0.094), mean ICF‐score (18, *p* = 0.045); Man.: mean UEL index (129.86, *p* = 0.240, mean ICF‐score (25, *p* = 0.001))	Across both groups: four episodes of erysipelas (*n* = 3; at 6 months)
Reilly et al. [18]	NA	NA	12	NA	NA

Abbreviations: LE, lower extremity; lymph‐ICF, lymphedema functioning; disability, and health questionnaire; man., manual; NA, not available; rob., robotic; ΔVol, volume reduction; UE, upper extremity; UEL, upper extremity Lymphedema.

Seven studies provided heterogeneous data on patient‐related outcomes: one study focused solely on the objective reduction of lymphedema [10], one study solely on subjective symptom regression [3], and five studies provided data on both the reduction of clinical manifestations such as lymphedema, lymphocele, anasarca, and chylothorax, as well as the regression of symptoms [1, 8, 15, 16, 17]. Volume measurement was used as an objective benchmark for the effectiveness of robotic‐assisted lymphatic reconstruction in two studies. In one of these, volume measurements were conducted at minimum 3 months postoperatively, whereby the following findings were observed: 86% of the arms (*n* = 6 out of 7) showed a volume reduction, with a mean reduction of 281 mL (7.6%), and in 72% of the legs (*n* = 23 out of 32), a volume reduction was achieved, with a mean reduction of 288 mL (1.4%) [16]. Furthermore, the study elaborated on one case in detail, in which a complete regression of symptoms was described 1 year postoperatively. The second study stated volume measurements solely in one case, in which a decrease of 25.2% in the upper extremity volume and, compared to the unaffected arm, a reduction of 100% in excess volume was achieved over a 3‐month period following robotic‐assisted gastroepiploic VLNT [10].

In the randomized controlled pilot trial, the mean upper extremity lymphedema (UEL) index, which is based on five circumference measurements at different sites of the affected arm, was recorded at baseline, 1 month, and 3 months to investigate the objective effects of robotic‐assisted and manual LVA [1]. The mean UEL index improved in the robotic‐assisted LVA group, while it increased in the manual LVA group over a period of 3 months postoperatively, as shown in Table 3. Yet, no significant changes in the mean UEL index between the groups at 1 and 3 months were detected. Further, analysis using a linear mixed model showed no significant intervention effect (comparing robotic‐assisted and manual LVA) at neither 1 month (−3.95, 95% CI −10.62 to 2.75, *p* = 0.230) or 3 months (−0.33, 95% CI −6.69 to 6.03, *p* = 0.914). Analogically, subjective effects were assessed by lymphedema functioning, disability, and health questionnaire (lymph‐ICF) total score at 1 and 3 months. Both the robotic‐assisted and manual LVA groups showed an improvement in mean percentage difference in lymph‐ICF total score of −41.17 in the robot‐assisted group and −41.57 in the manual LVA group (*p* = 0.98). However, differences in mean lymph‐ICF total score between the groups at 1 and 3 months were not significant. In addition, a linear mixed model analysis demonstrated that for the lymph‐ICF score, no significant difference was found when comparing robotic‐assisted and manual LVA at 1 month (8.31, 95% CI −6.75 to 23.37, *p* = 0.26) and 3 months (0.69, 95% CI −13.41 to 14.51, *p* = 0.92). In the 1‐year follow‐up, the mean UEL index did not decrease compared to the baseline in both the robotic‐assisted (*p* = 0.094) and manual LVA group (*p* = 0.240) [15]. In contrast, the lymph‐ICF total score improved significantly in both groups (robot‐assisted LVA, *p* = 0.045; manual LVA, *p* = 0.001).

In their case report, Lilja, Thomsen, and Sørensen described a complete remission of a lymphocele 14 days and 1 month postoperatively following its manual excision and robotic‐assisted LVA [17]. Further, the patient reported an improved control and functionality of his leg at the 1‐month follow‐up.

Both studies that focus on central lymphatic reconstruction analyzed subjective outcomes [3, 8], while only Weinzierl et al. reported an objective improvement of different clinical manifestations of lesions of the central lymphatic system, as discussed above.

The remaining four studies lack data on both the objective and subjective patient‐related outcomes.

Seven studies provided no data regarding complications. Among the other studies, one study reported different complications in patients following robotic‐assisted lymphatic reconstruction such as hematoma (*n* = 1) necessitating surgical evacuation and wound infections at the surgical site (*n* = 6), with two of these cases also exhibiting wound dehiscence and lymphocele which required surgical revision [16]. However, the authors mentioned that these complications occurred almost exclusively in patients receiving gastroepiploic VLNT to the ankle, thus leading to a revised surgical protocol, in which the recipient site was changed to the middle to lower leg. This resulted in a significantly improved wound healing.

Van Mulken et al. reported erysipelas infections (*n* = 2) following robotic assistance, which occurred during the 3‐month follow‐up period [1]. In the subsequent study describing the 1‐year outcome of the same patient cohort, four episodes of erysipelas in three patients were stated as complications after a 6‐month follow‐up period [15]. However, no specification was made on whether this referred to patients with robotic assistance or manual technique.

## 4 Discussion

This systematic review provides a comprehensive overview of the existing literature on peripheral and central robotic‐assisted lymphatic reconstruction, evaluating clinical outcomes, potential benefits, and future research directions. All 11 studies included confirmed the feasibility of robotic‐assisted lymphatic surgery and reported promising results concerning both technical aspects and patient‐related outcomes.

The advantages of microsurgical robotic platforms emphasized in the studies comprise enhanced precision through tremor filtering and motion scaling as well as facilitated access to deep‐lying anatomical structures. In addition to direct tremor filtering, the freestanding robotic system also provides the benefit of not causing any movement of the surgical field, as it occurs during manual techniques when the surgeon's hands are relocated [8]. This was highlighted to be especially advantageous in the anastomosis of vessels less than 1 mm and in the case of a very small operating field. Moreover, the Symani Surgical System allows teleoperative surgery, when it is combined with a 3D exoscope instead of a conventional microscope, thus leading to improved ergonomics and enabling a second surgical team to operate on the patient at the same time [2]. Further, teleoperation opens up the possibility that, in the distant future, patients undergo robotic‐assisted procedures from a different geographical location.

### 4.1 Temporal and Technical Aspects

A critical factor noted in the studies is the prolonged operation times associated with the use of microsurgical robots. These arise partly due to longer anastomotic times and partly due to the time required for setting up the robot and switching intraoperatively between manual and robotic‐assisted modalities [12]. However, it must be taken into account that most studies in this review presented their initial cases with robotic assistance, thus a decrease in mean robotic‐assisted anastomotic time can be expected for future studies conducted with more experience. Barbon et al. reported a significant learning curve for robotic‐assisted LVA, whereby the mean anastomotic time of the second patient group (16.3 ± 6.1 min) was comparable to manual anastomotic time (14.1 ± 4.3) [12]. In addition, nearly all other studies, which included more than one patient, also stated a decrease in anastomotic or total operation time with growing experience [1, 2, 10, 16, 18]. However, while Reilly et al. observed a reduction in the time required for preparing the robot, the time to perform a robotic‐assisted LVA did not decrease over time [18]. It should be noted that this study included only 12 patients; hence, it is possible that a learning curve would have been observed with a larger cohort.

Regarding the mean number of stitches per anastomosis, only one study compared the robotic‐assisted technique to the manual technique and found no significant difference in the average number of stitches between the two groups [12]. Furthermore, the weighted average of 6.49 stitches (range of mean number of stitches 4.5–7.9) for robotic‐assisted anastomoses across all studies is consistent with the numbers reported in the literature for manual microvascular anastomoses [19].

### 4.2 Clinical Outcomes

While all studies confirmed the feasibility of robotic‐assisted peripheral and central lymphatic reconstruction, only seven studies provided data on the objective or subjective improvement of the underlying condition. Hereby, heterogeneous measurement methods (volume vs. circumference measurement) and different protocols (e.g., regarding postoperative wearing of compression garment) were chosen, thus a direct comparison of the outcome of different studies was only possible to a limited extent, and solely descriptive statistics could be provided in this review. Van Mulken et al. statistically analyzed their data on the objective and subjective outcome and found a significant improvement in quality of life (QoL) in both the robotic‐assisted and manual groups in their 1‐year follow‐up study [15]. Further, all six remaining studies also reported encouraging postoperative outcomes, including volume reduction in limbs affected by lymphedema, improvement of anasarca and chylothorax, and complete regression of lymphocele in the groin, as well as regression of symptoms, even though these findings were not statistically analyzed.

### 4.3 Current Challenges

Despite the described benefits of microsurgical robotic platforms compared to manual techniques, there exist several challenges that need to be further evaluated to ensure an evidence‐based and effective implementation of robotic assistance in lymphatic reconstruction and plastic surgery in general.

As discussed in Section 4.1, the extended duration of robotic‐assisted procedures is a relevant drawback discussed in the majority of the studies included [1, 2, 10, 12, 14, 16, 18]. Another disadvantage highlighted in various studies is the additional costs that arise as a result of using a microsurgical robotic platform, which must be taken into account when specifying the indication of robotic assistance [3, 10]. This includes costs of acquisition, maintenance, consumables, and additional staff members needed during surgery, as well as indirect costs that result from longer operating times and training of surgeons and operating room staff [11]. In terms of preoperative training, it was reported that, despite their experience, the microsurgeons had to undergo several hours of training before performing in‐human robotic‐assisted anastomoses [1, 2, 14, 18]. Additionally, the lack of tactile feedback was listed as a potential disadvantage of robotic platforms in several studies included [2, 14, 18]. Yet, one study included stated that the absence of haptic feedback was well made up for by visual feedback, especially due to the similarity of the handling of the robot's manipulators and traditional microsurgical instrumentation [2]. Further, other studies investigating the use of robotic platforms in microsurgery also came to the conclusion that visual feedback could compensate for the absence of proprioceptive information [20, 21]. Lastly, Panchulidze et al. stated that microsurgeons do not rely on haptic feedback anyway when using 9‐0 or 10‐0 sutures, thus suggesting that this point of criticism may be less severe than originally assumed [22].

### 4.4 Limitations and Future Perspectives

Our review includes all available literature on robotic‐assisted lymphatic reconstruction. However, there are several limitations in this study due to the novelty of this field. To date, the majority of articles are retrospective feasibility studies with a limited number of participants and a lack of statistical analyses. Van Mulken et al. provided randomized data and conducted statistical analyses [1, 15]. Additionally, only three studies have contrasted the mean time for robotic‐assisted versus manual anastomoses, and only three studies (including the 1‐year follow‐up) have compared the patient‐related outcome between the two groups. Hereby, neither the microsurgeons nor the patients were blinded to the modality of the procedure (robotic‐assisted vs. manual), which may have led to a bias in the outcome. Further, five studies performed more than one type of surgical lymphatic intervention and two studies also conducted non‐lymphatic robotic‐assisted anastomoses aside from lymphatic reconstructive surgery [12, 14]. Another potential source of bias is the fact that multiple studies were conducted by the same centers, resulting in all 11 studies originating from just five different centers.

Future research in this field should aim to conduct randomized controlled studies with larger cohorts and extended follow‐up periods to statistically analyze the effect of robotic assistance in comparison to manual techniques and provide more robust and generalizable data. However, this could be especially difficult in central lymphatic reconstruction due to the rarity and individual character of these cases. In addition, standardization of the measurement protocols would lead to greater comparability of studies, thereby facilitating the drawing of conclusions regarding the advantages of robotic platforms versus manual methods, as well as allowing the comparison of the effect of different types of interventions. This would make it possible to define evidence‐based guidelines for robotic‐assisted lymphatic reconstruction.

## 5 Conclusion

This systematic review showed the feasibility and safety of using a dedicated microsurgical robotic platform in peripheral and central lymphatic reconstruction, which was confirmed in all 11 studies included. So far, there exist only a few studies directly comparing the robotic‐assisted approach to the manual approach with a limited number of statistical analyses; thus, a definitive statement on the possible superiority of robotic platforms is yet to be made. However, this review highlighted the promising potential of dedicated microsurgical robotic platforms in both central and peripheral lymphatic reconstruction and outlined the direction that future research in this field should follow to allow the drawing of evidence‐based conclusions.

## Conflicts of Interest

Nicole Lindenblatt acts as a scientific consultant and clinical advisor for Medical Microinstruments (MMI). The other authors declare no conflicts of interest.

## SYNOPSIS

We provide a systematic review of robotic‐assisted lymphatic surgery based on currently published literature. The literature search yielded 328 articles after the removal of duplicates, followed by a full‐text review of the 29 articles, out of which a total of 11 relevant articles were deemed eligible. Among these, seven used a retrospective design and four a prospective design. Despite the heterogeneous measurands, all studies showed the feasibility of robotic‐assisted lymphatic surgery, and seven provided promising data on the patient‐related outcomes. Additional studies are needed to further identify future directions in robotic‐assisted lymphatic surgery.

## Supporting information

Supporting information.

## Data Availability

Research data are not shared.
