# Supplementary material for: Systematic Review of Robotic‐Assisted Peripheral and Central Lymphatic Surgery
Source: J Surg Oncol. 2024 Sep 10;131(1):62–71. doi: 10.1002/jso.27866 (PMC11874190; doi:10.1002/jso.27866)
Supplement: Supplementary file 1 — Supporting information. [file JSO-131-62-s001.docx]

**Appendix: Literature search strategy, 4th June 2024**

| Database: Embase via Elsevier | | |  |
| --- | --- | --- | --- |
| No. | **Query** | **Results** | |
| #5 | #4 NOT (('animal'/de OR 'animal experiment'/exp OR 'nonhuman'/de) NOT ('human'/exp OR 'human experiment'/de)) | | 172 |
| #4 | #3 AND ([english]/lim OR [german]/lim) | | 177 |
| #3 | #1 AND #2 | | 181 |
| #2 | 'lymphovenous anastomosis'/exp OR 'lymphedema'/exp/mj OR (('lymph vessel'/exp OR 'lymphoid tissue'/de) AND ('reconstructive surgery'/exp OR 'anastomosis'/exp OR 'microvascular surgery'/exp)) OR ('lymph node'/exp/mj AND 'transplantation'/exp) OR (((lymphedem* OR lymphoedem* OR lymphovascular OR lymphat* OR microlymphat* OR 'lymph node' OR lymphnode OR lympho*venous OR 'lympho venous' OR lympholymphat* OR 'thoracic duct vein' OR 'thoracic duct-to-vein') NEAR/3 (surgery OR anastomosis OR reconstruct* OR bypass OR shunt OR transplant* OR transfer OR flap OR lva OR lnva OR vlnt OR ltt OR tdva)):ti,ab,kw) | | 32109 |
| #1 | 'robot assisted microsurgery'/exp OR 'robot assisted surgery'/de OR 'robotic surgical device'/exp OR ((robot* NEAR/3 (surg* OR microsurg* OR supermicrosurg* OR platform* OR reconstruct*)):ti,ab,kw) OR (robot*:ti,ab,kw AND (symani:ti,ab,kw OR musa:ti,ab,kw OR 'musa 2':ti,ab,kw OR 'musa 3':ti,ab,kw OR microsure:ti,ab,kw OR 'medical microinstruments':ti,ab,kw)) | | 50916 |

| Database: Medline via Ovid | |  |
| --- | --- | --- |
| No. | **Query** | **Results** |
| 1 | (Robotics/ and (Surgical Procedures, Operative/ or Microsurgery/ or Plastic Surgery Procedures/)) or Robotic Surgical Procedures/ or (robot* adj3 (surg* or microsurg* or supermicrosurg* or platform* or reconstruct*)).ti,ab,kw. or (robot* and (Symani or MUSA or MUSA-2 or MUSA-3 or Microsure or "medical microinstruments")).ti,ab,kw. | 30054 |
| 2 | exp *Lymphedema/ or ((exp Lymphatic Vessels/ or Lymphoid Tissue/) and (Surgical Procedures, Operative/ or Plastic Surgery Procedures/ or Anastomosis, Surgical/ or Microsurgery/)) or (exp *Lymph Nodes/ and exp Transplantation/) or exp Lymph Nodes/tr or ((lymphedem* or lymphoedem* or lymphovascular or lymphat* or microlymphat* or "lymph node" or lymphnode or lympho*venous or "lympho venous" or lympholymphat* or "thoracic duct vein" or "thoracic duct-to-vein") adj3 (surgery or anastomosis or reconstruct* or bypass or shunt or transplant* or transfer or flap or LVA or LNVA or VLNT or LTT or TDVA)).ti,ab,kw. | 17213 |
| 3 | 1 and 2 | 96 |
| 4 | limit 3 to (english or german) | 94 |
| 5 | 4 not (animals not humans).sh. | 94 |

| Database: Cochrane | |  |
| --- | --- | --- |
| No. | **Query** | **Results** |
| 1 | (robot* NEAR/3 (surg* OR microsurg* OR supermicrosurg* OR platform* OR reconstruct*)):ti,ab,kw OR (robot* AND (Symani OR MUSA OR MUSA-2 OR MUSA-3 OR Microsure OR "medical microinstruments")):ti,ab,kw | 2715 |
| 2 | ((lymphedem* OR lymphoedem* OR lymphovascular OR lymphat* OR microlymphat* OR "lymph node" OR lymphnode OR lympho*venous OR "lympho venous" OR lympholymphat* OR "thoracic duct vein" OR "thoracic duct-to-vein") NEAR/3 (surgery OR anastomosis OR reconstruct* OR bypass OR shunt OR transplant* or transfer OR flap OR LVA OR LNVA OR VLNT OR LTT OR TDVA)):ti,ab,kw | 1329 |
| 3 | #1 AND #2 | 71 |
| 4 | Limit #4 to English | 67 |

| Database: Web of Science Core Collection | |  |
| --- | --- | --- |
| No. | **Query** | **Results** |
| 1 | TS=(robot* NEAR/3 (surg* OR microsurg* OR supermicrosurg* OR platform* OR reconstruct*)) OR TS=(robot* AND (Symani OR MUSA OR MUSA-2 OR MUSA-3 OR Microsure OR "medical microinstruments")) | 39088 |
| 2 | TS=((lymphedem* OR lymphoedem* OR lymphovascular OR lymphat* OR microlymphat* OR "lymph node" OR lymphnode OR lympho*venous OR "lympho venous" OR lympholymphat* OR "thoracic duct vein" OR "thoracic duct-to-vein") NEAR/3 (surgery OR anastomosis OR reconstruct* OR bypass OR shunt OR transplant* or transfer OR flap OR LVA OR LNVA OR VLNT OR LTT OR TDVA)) | 6223 |
| 3 | #1 AND #2 | 123 |
| 4 | #1 AND #2 and English or German (Languages) | 121 |

| Database: Web of Science Preprint Citation Index | |  |
| --- | --- | --- |
| No. | **Query** | **Results** |
| 1 | TS=(robot* NEAR/3 (surg* OR microsurg* OR supermicrosurg* OR platform* OR reconstruct*)) OR TS=(robot* AND (Symani OR MUSA OR MUSA-2 OR MUSA-3 OR Microsure OR "medical microinstruments")) | 1290 |
| 2 | TS=((lymphedem* OR lymphoedem* OR lymphovascular OR lymphat* OR microlymphat* OR "lymph node" OR lymphnode OR lympho*venous OR "lympho venous" OR lympholymphat* OR "thoracic duct vein" OR "thoracic duct-to-vein") NEAR/3 (surgery OR anastomosis OR reconstruct* OR bypass OR shunt OR transplant* or transfer OR flap OR LVA OR LNVA OR VLNT OR LTT OR TDVA)) | 7 |
| 3 | #1 AND #2 | 0 |

| Database: LILACS via VHL Regional Portal | |  |  |
| --- | --- | --- | --- |
| No. | **Query** | **Results** | **Comments** |
| 1 | ((robot* AND (surg* OR microsurg* OR supermicrosurg* OR platform* OR reconstruct* OR symani OR musa OR musa-2 OR musa-3 OR microsure OR "medical microinstruments"))) AND (((lymphedem* OR lymphoedem* OR lymphovascular OR lymphat* OR microlymphat* OR "lymph node" OR lymphnode OR lympho*venous OR lympholymphat* OR "thoracic duct vein" OR "thoracic duct-to-vein") AND (surgery OR anastomosis OR reconstruct* OR bypass OR shunt OR transplant* OR transfer OR flap OR lva OR lnva OR vlnt OR ltt OR tdva))) | 4751 | Search in Title, abstract, subject |
| 2 | #1 AND ( db:("LILACS")) | 30 |  |
| 3 | #2 AND la:("en") | 21 |  |

| Trial register: US Clinical trials register via clinicaltrials.gov | |  |
| --- | --- | --- |
| Search field | **Query** | **Results** |
| Intervention/ treatment | (Robot AND microsurgery) OR Symani OR Microsure OR “Medical microinstruments” | 3 |
| Title and/or Title Acronym | Lymphatic OR thoracic | 2812 |
|  | Combined with AND | 0 |

| Trial register: WHO International Clinical Trials Registry Platform via Search Portal | |  |
| --- | --- | --- |
| Search field | **Query** | **Results** |
| Intervention | (robot* AND (surg* OR microsurg* OR supermicrosurg* OR anastom* OR platform* OR reconstruct* OR bypass OR shunt OR transplant* OR transfer OR flap OR LVA OR LNVA OR VLNT OR LTT OR TDVA OR Symani OR MUSA OR MUSA-2 OR MUSA-3 OR Microsure OR "medical microinstruments")) | 199 |
| Title | Lymphatic OR thoracic | 5997 |
|  | Combined with AND | 10 |
